# Replicatively senescent cells are arrested in G1 and G2 phases

**DOI:** 10.18632/aging.100467

**Published:** 2012-06-28

**Authors:** Zhiyong Mao, Zhonghe Ke, Vera Gorbunova, Andrei Seluanov

**Affiliations:** Department of Biology, University of Rochester, Rochester NY 14627

**Keywords:** replicative senescence, aging, cell cycle, human fibroblasts

## Abstract

Most human somatic cells do not divide indefinitely but enter a terminal growth arrest termed replicative senescence. Replicatively senescent cells are generally believed to arrest in G1 or G0 stage of the cell cycle. While doing cell cycle analysis on three different lines of normal human fibroblasts we observed that 36-60% of the replicatively senescent cells had 4N DNA content. Only up to 5% of senescent cells had more than one nucleus ruling out the possibility that the 4N cell population were G1-arrested bi-nucleated cells. Furthermore, it is unlikely that the 4N cells are tetraploids, because actively dividing pre-senescent cultures lacked the 8N tetraploid G2 population. Collectively these results suggest that the 4N population consists of G2 arrested cells. The notion that a large fraction of senescent cell population is arrested in G2 is important for understanding the biology of replicative senescence.

## INTRODUCTION

The phenomenon of replicative senescence has been described in 1961 by Hayflick and Moorhead [1], and has since been actively investigated by biogerontologists. Senescent cells were later found to affect surrounding cells by secreting inflammatory cytokines [2] and were recently shown to promote age-related pathology in vivo [3]. Replicatively senescent cells are believed to arrest in G1 or G0 stage of the cell cycle. Interestingly, early studies performed in 1970th noted that populations of replicatively senescent cells contained a sizable fraction of cells with 4N DNA content [4-6]. It was not possible at that time to conclusively distinguish G2 cells from tetraploid G1 cells. In some reports it was speculated that these cells were tertaploids arrested in G1 [4, 6]. Later these studies forgotten and G1/G0 arrest in senescent cells became a dogma.

## RESULTS AND DISCUSSION

**Figure 1:**
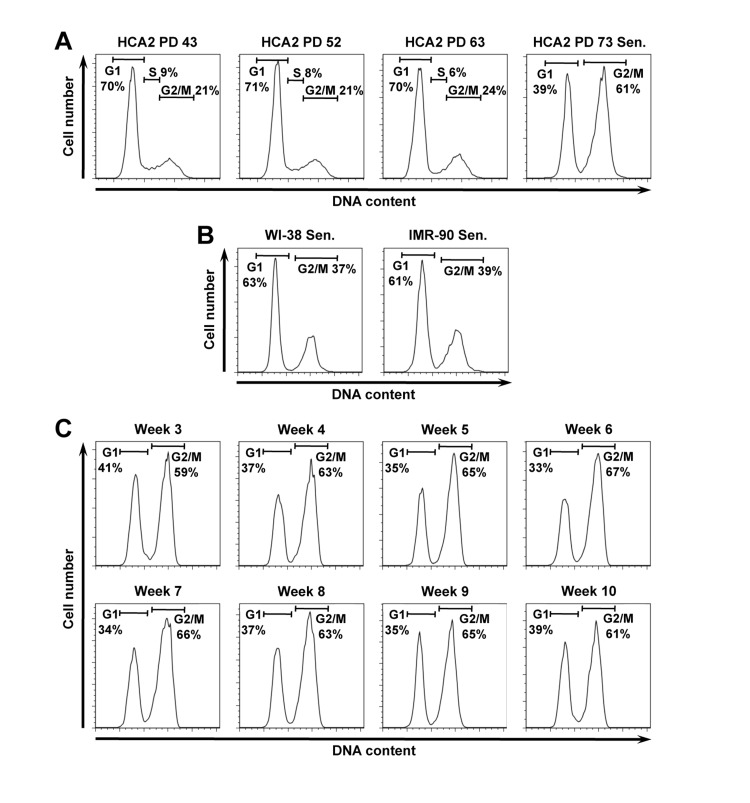
Senescent human fibroblast cultures contain a large fraction of putative G2-arrested cells with 4N DNA content. (**A**) Propidium iodide (PI) staining and flow cyctometric analysis of HCA2 normal human foreskin fibroblasts. Cells entered senescence at PD73. (**B**) PI staining of replicatively senescent human lung fibroblasts WI-38, and IMR-90 at PDs 73 and 68 respectively. (**C**) The fraction of 4N cells in senescent cell population does not diminish with time. Replicatively senescent HCA2 cells were analyzed by PI staining at weekly intervals for 10 weeks stating from the onset of senescence.

We observed that 60% of the population of replicatively senescent normal human foreskin fibroblasts HCA2 consisted of cells with 4N DNA content (Figure 1A), while the remaining cells were in G1 stage. Intrigued by the result we repeated propidium idodide (PI) staining on replicatively senescent WI-38 and IMR-90 cells, which are the two commonly used normal human fibroblast strains. The senescent WI-38 and IMR-90 cells contained 37% and 39% of cells with 4N DNA content, respectively (Figure 1B).

We hypothesized that these cells are transiently arrested in G2 stage and have not yet progressed to mitosis. We then incubated HCA2 senescent cells for additional 10 weeks counting from the time the cultures entered senescence and proliferation ceased and repeated cell cycle analysis weekly. No changes in cell cycle distribution were observed (Figure 1C). This result suggests that the observed cell cycle distribution with 2N and 4N populations is a stable terminally growth arrested state for the human fibroblasts.

**Figure 2:**
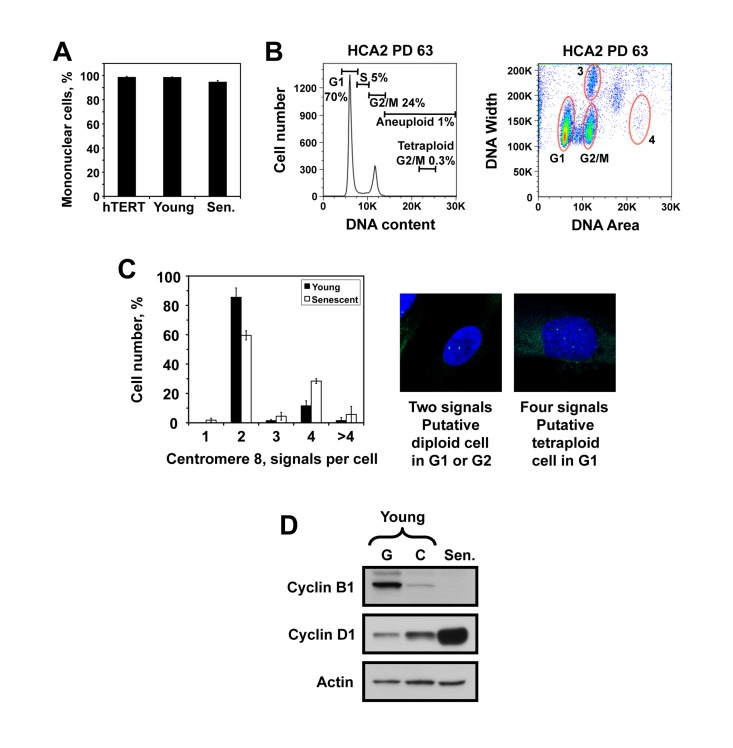
The senescent cell population with 4N DNA content is not due to polynucleated cells or tetraploid cells. (**A**) The number of nuclei per cell was counted in hTERT-immortalized, young, and senescent HCA2 cells after staining with DAPI and tubulin. (**B**) PI staining and flow cytometric analysis of pre-senescent HCA2 cells showing the absence of tetraploid G2 population. The left panel is the conventional DNA content histogram, and the right panel is a corresponding area versus width plot. Zone 3 contains two-cell aggregates of cells with 2N DNA content, and zone 4 shows a putative position of tetraploid G2 cells with 8N DNA content. (**C**) Fluorescent in situ hybridization with a probe to chromosome 8 centromeric region in young and senescent HCA2 cells. Diploid G1 or G2 cells show two signals, while tetraploid G1 cells are expected to show four signals. Hypothetically, if sister chromatids separate after a prolonged G2 arrest a diploid G2 cells may also show four signals. The percentages of cells with indicated numbers of chromosome 8 centromeric signals are plotted. The experiment was repeated three times and error bars show s.d. (**D**) Cyclin levels in replicatively senescent cells. Western blot of total cell lysates from HCA2 cells probed with cyclin B1 (Abcam, ab72-100) and cyclin D1 (Abcam, ab10540-100) antibodies.

Although the cell cycle distributions we observed (Figure 1) closely resembled G1/G2 population, we considered two alternative explanations for the origin of the 4N cells. First, these cells may be polynucleated, containing two nuclei with 2N DNA content. Microscopy analysis of senescent cells stained with DAPI showed that senescent cell population contained 5% of cells with more than one nucleus (Figure 2A), which cannot account for the observed 36-60% of cells with 4N DNA content. The second possibility is that the 4N cells are tertaploid cells in G1 stage. This implies a very high level of polyploidy in replicatively senescent fibroblasts. To test this possibility, we examined presenescent HCA2 cells at PD63, which are still actively dividing, for the presence of tertaploid G2 fraction with 8N DNA content. The fraction of such cells was 0.3%, indicating that the presenescent culture contains a very low number of tetraploid cells (Figure 2B). This result argues against the hypothesis that 4N population observed in senescent cultures consists of G1 arrested tetraploids. We then performed in situ staining of young and replicatively senescent HCA2 cells with a probe to the centromeric region of chromosome 8. Diploid G1 cells are expected to show two signals corresponding to the two homologues of chromosome 8, diploid G2 cells are also expected to show two signals corresponding to two pairs of closely positioned sister chromatids, while tetraploid G1 cells are expected to show four signals. Eighty five percent of young cells showed two signals, and 11% showed four signals. Sixty percent of senescent cells showed two signals, and 28% showed four signals (Figure 2C). As described above, HCA2 senescent culture contained 40% of cells with 2N DNA content and 60% of cells with 4N DNA content (Figure 2B), thus tetraploid G1 cells can potentially explain only less than half of the population of cells with 4N DNA content. Combined with the lack of tetraploid G2 population in pre-senescent cultures (Figure 2B), this result indicates that the 4N cell population is unlikely to be composed of G1-arrested tetraploid cells. To explain the origin of the cells with four dispersed centromeres, we hypothesize that when senescent cells undergo prolonged arrest in G2 stage, sister chromatids may separate resulting in appearance of cells with 4N DNA content and four separated chromatids. We further analyzed the protein level of cyclins in replicatively senescent cells. In agreement with previous findings [7-9], the level of cyclin D1 which promotes progression through G1/S phases was elevated, while cyclin B1, promoting G2/M transition, was absent in senescent cells (Figure 2D). This cyclin profile is consistent with G2-arrest.

Collectively, our results indicate that replicatively senescent cell populations arrest in both G1 and G2 stages. One potential implication of G2 arrest in senescent cells is that many of these cells retain sister chromatids available as a backup or as a DNA repair template. Another implication is that the 4N DNA content may contribute to the complex changes in expression patterns observed in senescent cells.

It was proposed that replicative senescence represents a state where the growth-promoting TOR pathway is activated, while the cell cycle is blocked leading to cellular hypertrophy and the typical enlarged phenotype of senescent cells [10]. Indeed, treatment with TOR inhibitor rapamycin suppressed senescence in mouse cells [11], and inhibition of TORC1 attenuated replicative and RAS-induced senescence in human cells [12]. Our finding that a large fraction of replicatively senescent cells arrests in G2 phase supports this view of replicative senescence. We propose that as cells enter replicative senescence they progress to G2 phase due to activated TOR pathway but cannot enter to mitosis. As a result, replicatively senescent cells accumulate in G2 phase, and a mixture of G1/G2 – arrested cells represents the terminal cell cycle arrest in replicatively senescent cultures.

## METHODS

### Cell lines and culture conditions

IMR-90 and LF1 are normal human lung fibroblasts. WI-38 fibroblasts and IMR-90 cells were from the Coriell institute for Medical research, and HCA2 human foreskin fibroblasts were a kind gift from Olivia Pereira-Smith. Cells were grown in Eagle's minimum essential medium (ATCC, 30-2003) supplemented with 15% fetal calf serum (Gibco, 10437-028) and 1% penicillin-streptomycin (ATCC, 30-002).

### Cell cycle analysis

Cell cycle distribution was examined with propidium iodide (PI) staining. Cells were harvested and fixed with 70% ethanol for at least 16 hours. After fixation, cells were washed twice with 1×PBS, followed by incubation with 1ml 1×PBS containing 20μg/ml PI and 1mg/ml RNase A for 30 minutes at room temperature. PI stained samples were analyzed on the FACSCantoII machine (BD Biosciences). At least 10,000 events were collected in each analysis. Data was further analyzed by Flowjo software.

### Immunofluorescence

Cells were fixed with 4% paraformaldehyde at 254°C for 15 minutes, followed by 3 washes with 1×PBS. Cells were permeabilized with 0.25% Triton X-100 for 10 minutes and washed with ice-cold 1×PBS three times. After blocking with 1% BSA for 1 hour at room temperature, cells were incubated with anti-β tubulin (1:500, Abcam, ab6046) overnight at +4°C. Cells were washed with 1×PBS three times, followed by a one hour incubation with goat-anti-rabbit-FITC (1:500, Abcam, ab6717) at 25°C. Cells were then washed with 1×PBS three times, and incubated with 1μg/ml DAPI for 2 minutes, followed by three quick washes with 1×PBS. Then, the samples were covered with mounting medium (Vector Laboratory, H-1000).

### Fluorescence In Situ Hybridization

Cells were fixed with 2×SSC containing 2% formaldehyde for 15 minutes at room temperature. After the cells were washed three times with PBS, the fixation was stopped by incubating cells with 2 mg/ml glycine for 10 minutes at room temperature, followed by another three washes with PBS. Then slides were sent out for FISH experiment using a probe against the centromere of chromosome 8. Staining was performed by the Cytogenetic Center at the University of Rochester.
